# Ranking of Production Animal Welfare and Ethics Issues in Australia and New Zealand by Veterinary Students

**DOI:** 10.3390/vetsci5030065

**Published:** 2018-07-12

**Authors:** Amelia Cornish, Andrew D. Fisher, Teresa Collins, Chris Degeling, Rafael Freire, Susan J. Hazel, Jennifer Hood, Janice K. F. Lloyd, Clive J. C. Phillips, Kevin J. Stafford, Vicky Tzioumis, Paul D. McGreevy

**Affiliations:** 1 Sydney School of Veterinary Science, University of Sydney, Sydney, NSW 2006, Australia; jenih@iinet.net.au (J.H.); vicky.tzioumis@sydney.edu.au (V.T.); paul.mcgreevy@sydney.edu.au (P.D.M.); 2 Faculty of Veterinary and Agricultural Sciences, University of Melbourne, Parkville, VIC 3010, Australia; adfisher@unimelb.edu.au; 3 School of Veterinary and Life Science, Murdoch University, Murdoch, WA 6150, Australia; t.collins@murdoch.edu.au; 4 Research for Social Change, Faculty of Social Science, University of Wollongong, Wollongong, NSW 2522, Australia; degeling@uow.edu.au; 5 School of Animal and Veterinary Science, Charles Sturt University, Wagga, NSW 2650, Australia; rfreire@csu.edu.au; 6 School of Animal and Veterinary Science, University of Adelaide, Roseworthy, SA 5005, Australia; susan.hazel@adelaide.edu.au; 7 College of Public Health, Medical and Veterinary Science, James Cook University, Townsville, QLD 4811, Australia; janice.lloyd@jcu.edu.au; 8 School of Veterinary Science, University of Queensland, Gatton, QLD 4343, Australia; c.phillips@uq.edu.au; 9 Institute of Veterinary, Animal and Biomedical Science, Massey University, Palmerston North 4442, New Zealand; k.j.stafford@massey.ac.nz

**Keywords:** animal welfare, veterinary education, Day One competence, gender, production animals, veterinary ethics, euthanasia

## Abstract

The importance of animal welfare and ethics (AWE) within the veterinary education should reflect community concerns and expectations about AWE, and the professional demands of veterinary accreditation on the first day of practice (or ‘Day One’ competences). Currently, much interest and debate surrounds the treatment of production animals, particularly around live export. To explore the attitudes to AWE of veterinary students in Australia and New Zealand, a survey was undertaken to (i) understand what students consider important AWE topics for initial production animal competence; and (ii) ascertain how these priorities correlated with gender, area of intended practice and stage-of-study. The results from 575 veterinary students showed that all students ranked strategies to address painful husbandry procedures as the most important issues on their first day in production animal practice. Additionally, it was found that the importance students assigned to an understanding of human–animal interactions declined as they progressed through the veterinary course. In contrast, the importance of an understanding of euthanasia issues for production animals increased for male students as they progressed through the course, and remained consistently high in females. Females also gave higher ranking to the importance of understanding production animal stress associated with transport, and ranked strategies to address painful husbandry procedures more important than did males. These findings should help the development of AWE teaching resources that address students’ attitudes and competence and that can be delivered when students are most receptive.

## 1. Introduction

There is a widely recognized need to ensure that the animal welfare knowledge of newly graduated veterinarians reflects the growing interest and demands of contemporary society in relation to animal welfare and ethics (AWE) [1,2]. In addition to directly influencing animal care and having a professional obligation to protect the welfare of animals [3], veterinarians are viewed as a trusted source of information and expertise on animal welfare matters [4].

The welfare of production animals is one of the cornerstones of public interest in animal welfare, with common concerns about farm animal welfare including issues such as restriction of movement, invasive husbandry practices that may cause pain, long-distance transport, and challenges that may be induced by the production environment [5]. However, previous research has indicated that veterinary students’ attitudes toward production animals’ cognitive abilities and sensitivity to pain may be less developed than their attitudes towards companion animals, moreover that their empathy and concern for animal welfare in general may decline during the veterinary course, and that the attitudes of male and female students may differ markedly [6,7]. Veterinary students at two universities in the United Kingdom were surveyed by Paul and Podberscek [6], who reported that students in later years of the program had reduced empathy and assigned less sentience to farm animals, notably cattle, and that this decline was largely driven by lower and declining scores from male students compared with those from female students. Drawing data from students at a single veterinary college in the United States, Levine et al. [8] found that veterinary students viewed cattle and small ruminants as having lesser cognitive capacity than dogs and cats, and that veterinary students who planned to work in production animal practice accepted a wider range of husbandry practices as being humane than did students aspiring to work as companion animal veterinarians. In a study of students at a European veterinary school, Kielland et al. [9] found that clinical conditions such as mastitis and pelvic fractures in cattle were rated as less painful by male students than by female students, and that the severity of assessed pain was lower when scored by students in later years of the veterinary course.

In response to these potential challenges and needs relating to production animal welfare knowledge and attitudes, veterinary educators need to be aware of the growing importance of AWE education for veterinary students. An important concept in veterinary education is that students should attain Day One competences that reflects the knowledge, skills, and attitudes expected for graduates to have on their first day of practice [10], many of which relate to AWE. To examine how veterinary students across all veterinary schools in Australia and New Zealand viewed production AWE issues in relation to Day One competences we surveyed students, both male and female, and at different stages of their course. The survey was designed to: (i) reveal what veterinary students in Australia and New Zealand consider to be important AWE topics for students’ initial competence in production animal matters; and (ii) study how selection of these priorities aligns with published evidence on the effects of gender, area of intended practice, and stage-of-study.

## 2. Materials and Methods

### 2.1. Study Participants

All students enrolled in veterinary science or veterinary medicine courses at all universities in Australia and New Zealand during October 2014 were invited to participate in the survey. Data from 575 students are explored in more details within. Institutional human ethics approval was granted by the University of Sydney Human Ethics Committee and by participating universities prior to the start of data gathering (approval number: 2014/739).

### 2.2. The Questionnaire

AWE teaching academics from each of the veterinary schools in Australia and New Zealand came together for a 2-day workshop, which was held at The University of Sydney in April 2014. This event provided a unique opportunity for attendees to use their collective expertise in all areas of AWE teaching and academia to discuss and rank, based on group consensus, a list of AWE questions and topics, which then became the content for the questionnaire.

A quantitative questionnaire was designed to explore the opinions of students regarding their priorities for the agreed AWE topics. SurveyMonkey (www.surveymonkey.com) was used to administer the online questionnaire from 9 October to 14 November 2014. Voluntary participation of students was sought via three e-mails sent a week apart, each of which included a link to the questionnaire. The link was closed approximately one week after the final reminder email (see Table 1). An award of AUD $200 to the representative student body at the institution with the highest participation rate provided an incentive for students to complete the survey.

The first four questions concerned consent to participate and student demographics (i.e., university, gender, and year-of-study) and were multiple-choice with single answer. Students were then asked to identify the type of work they expected to undertake on graduation. The survey then presented the RCVS Day One Competences, listing the skills and knowledge students should expect to have on the first day of practice. The questions on production animals required students to use a ten-point Likert scale to indicate from extremely important (1) to least important (10), how important an understanding of each AWE topic was for veterinarians on their first day of practice with production animals. The following list of production AWE topics were considered of most importance by consensus within the authors and presented for the students’ consideration: (i) Ethics of sustainable production (food security and animal welfare issues); (ii) Human–animal interactions and impacts on animals; (iii) Intensive versus extensive production systems; (iv) Slaughter and pre-slaughter inspections; (v) Social, economic, and cultural drivers of welfare outcomes; (vi) Strategies to address painful husbandry procedures; (vii) Distress associated with road, sea, and air transport; and (viii) Euthanasia.

### 2.3. Data Management

Given some differences in course structure across the universities surveyed, the responses to the question that asked students to identify their year of study were recoded with Years 1 and 2 as Early students, Years 3 and 4 as Mid-stage students, and Years 5 and 6 as Senior students. The question that asked students the type of work they expected to undertake on graduation permitted one or more responses and, therefore, the percentage of students reporting an interest in a particular type of work was expressed as a percentage of the total number of responses.

### 2.4. Statistical Analysis

All data were checked for errors and the cleaned data were entered into GenStat Version 15 (VSN International, Hemel Hempstead, UK). Log-linear modelling was used to analyse the three-way contingency tables of frequencies associated with (i) gender and (ii) stage-of-study. In the analysis, *p* < 0.05 was considered statistically significant. Log-linear modelling relies on expected frequencies that are not too small, generally not less than 1. Given that there were fewer than 20 male respondents from the senior years of study across all universities, we needed to apply caution where frequencies fell below 5 and, to address loss of statistical power, scores of 6 or more were combined for the purposes of analysis, but plots in this report are based on the percentages of all scores.

## 3. Results

### 3.1. Student Demographics

Of the 3320 students invited via email, 818 participated in the voluntary survey, and 575 students provided responses on production animal questions, the responses of these 575 students responses are discussed in more detailed below (Table 1). There were 673 (82%) females and 145 (18%) males. The proportion of students who expressed a desire to work after graduation in production animal practice was 10.0%, but a further 30.1% of respondents expressed a desire to work in mixed practice. The other preferred occupations nominated by students were companion animal practice (25.2%), exotic animal practice (9.4%), equine practice (7.6%), research (5.1%), government work (5.4%), and ‘don’t know’ (4.8%).

### 3.2. Student-Ranked Importance of Topics

**Figure 1:**
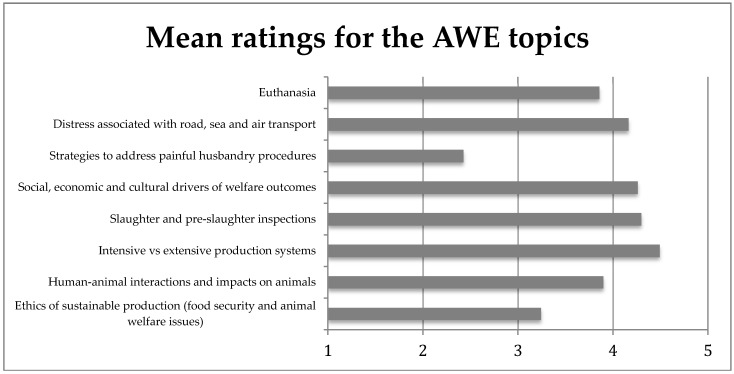
Mean ratings (1 = extremely important, 10 = least important) in answer to the question “How important is an understanding of the following topics for veterinarians on their first day in practice?”.

Students ranked strategies to address painful husbandry procedures as the most important AWE topic on their first day in production animal practice (Figure 1). Differentiating between intensive and extensive animal systems in terms of their effects of animal welfare was ranked as the least important topic for a newly graduated veterinarian. Notably, students ranked all the identified AWE issues in production animals as being of at least moderate importance (with a mean score of 5 or less).

### 3.3. Effect of Stage-of-Study and Gender on Student-Ranked Importance of Topics

**Figure 2:**
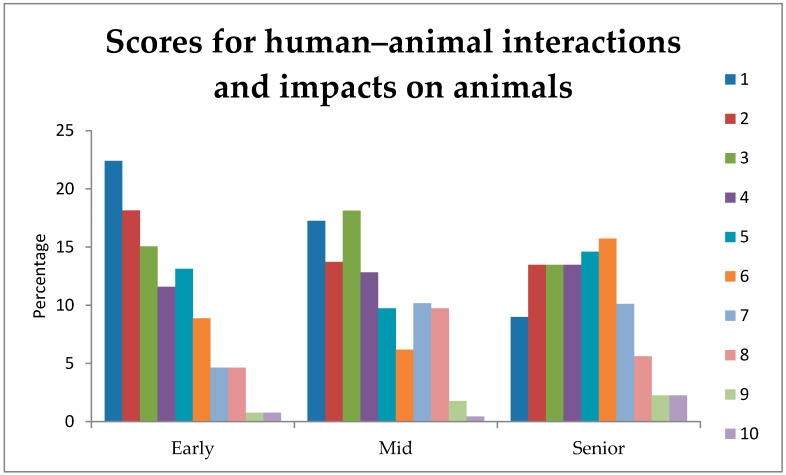
Percentage of students from the Early, Mid, and Senior stages-of-study rating (1 = extremely important, 10 = least important) of the importance of an understanding of human–animal interactions and impacts on production animals on their first day in practice.

The overall effects of stage-of-study, gender, and their interaction are presented in Table 2. Human–animal interactions and associated impacts on production animals were significantly influenced by stage-of-study, being ranked as more important by Early students, lower by Mid-stage students, and lower still by Senior students (Figure 2), with mean ranking scores of 3.53, 4.06, and 4.56, respectively (*p* = 0.03). The full results from the log-linear models will be provided upon request to the authors.

**Figure 3:**
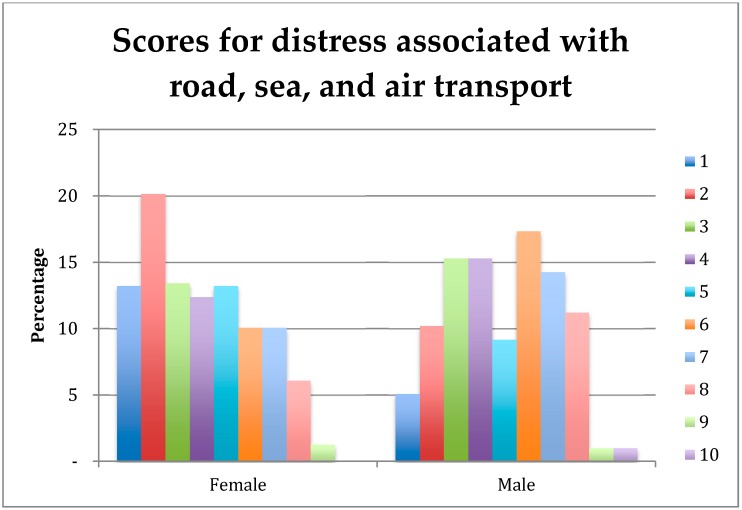
Percentage of female (*n* = 671) and male (*n* = 145) students rating (1 = extremely important, 10 = least important) of the importance of understanding distress associated with road, sea, and air transport on their first day in practice.

There was a significant effect of gender on student rankings of the importance of distress associated with road, sea and air transport (Figure 3). Female students, independent of stage-of-study, ranked this topic as being more important than did male students (4.01 versus 4.92, respectively; *p* = 0.001).

**Figure 4:**
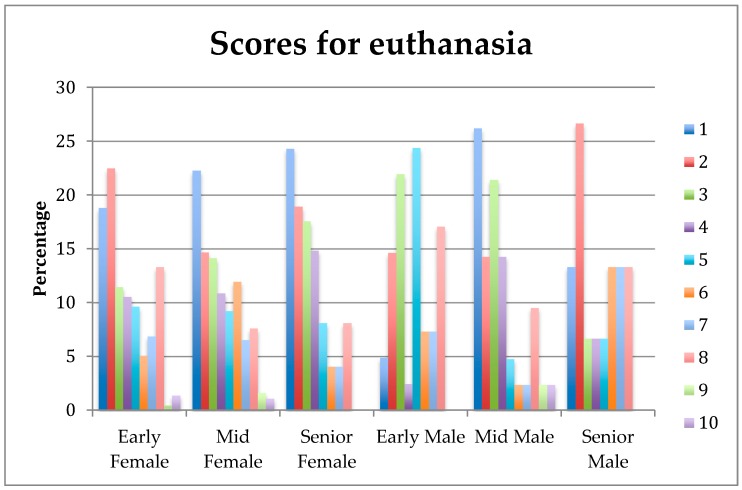
Percentage scores for female (*n* = 671) and male (*n* = 145) students from the Early, Mid, and Senior stages-of-study rating (1 = extremely important, 10 = least important) of the importance of their understanding of euthanasia of production animals on their first day in practice.

There was an interaction between gender and stage-of-study on the rankings for the importance of euthanasia as a Day One competence in production animals (Figure 4), in that female students ranked this question consistently higher throughout all stages of the course, whereas male students ranked it lower in the early and senior stages of the course (*p* = 0.02).

## 4. Discussion

The main findings of this study were that students ranked strategies to address painful husbandry procedures as the most important AWE topic on their first day in production animal practice. These findings suggest that students will embrace strategies to reduce the impact of invasive husbandry techniques as they progress from being students to working professionals. However, a counter-point may be that these findings suggest that students are merely more aware of negative welfare associated with painful husbandry in production animal management and that they consider their future role in ensuring good welfare as simply involving reducing and relieving pain. Such an interpretation may also be reinforced by our current finding that topics with wider implications and additional stakeholders involved, such as ‘Distress associated with road, sea, and air transport’ were ranked fairly poorly. Given current media and public scrutiny of live export, the absence of live export as a feature of the current findings may be surprising, However, it is worth noting that the survey was undertaken in 2014.

The differences identified between female and male student responses in this study partially align with the findings of previous studies that have identified consistently higher levels of empathy in relation to animal welfare among female students. Paul and Podberscek [6] found that female veterinary students expressed stronger beliefs about the impact of pain on the welfare of cows, and had higher rankings for overall cow sentience. Similarly, Kielland et al. [9] found that conditions, such as mastitis and pelvic fractures, in cattle were rated as less painful by males than by female students. Interestingly, in the current study, a significant effect of gender was found for only student rankings of the importance of distress associated with road, sea, and air transport. This may mean that female students show more empathy than males. Given the wealth of literature indicating that females show more concern for animal welfare and empathy for animals in general, these findings are noteworthy. However, one of the contemporary challenges in undertaking surveys of veterinary students to examine gender differences is the relative scarcity of male students. In the current study, the proportion of male students was 17% (*n* = 98), which was low, but reflective of the percentage enrolled in the veterinary course across Australia and New Zealand.

Although other studies have reported that students in later years of the veterinary course rated animal welfare issues as less important than early-stage students did [11], the results of our study varied according to the topic being examined. Human–animal interaction and its importance for animal welfare was the only topic that declined in importance among later stage students. This may reflect that this fundamental aspect of animal welfare science is typically presented early in the veterinary curriculum and that the focus of later-stage students shifts to what they perceive they will need in terms of clinical skills to promote animal welfare when they graduate. Martin et al. [12] similarly found that the importance of the human–animal bond to students decreased over the veterinary course and suggested that students perceive a need to maintain a ‘professional distance’ in the field, which may also be partly responsible for their assigning less importance to the human–animal bond as they progressed through the course. Given the widely demonstrated benefits of positive human–animal interactions for production animal productivity and health [13,14,15], it may be worthwhile re-emphasising this concept by embedding it into clinical teaching in production animal health.

In the present study, the importance of euthanasia as a Day One competence for production animals increased in the later stages of the course for male students, but was consistently high for female students. Although the topics of euthanasia and animal ethics are not perfectly aligned, an earlier study by Verrinder and Phillips [16] found that both early and late-stage veterinary students indicated strong concern for animal ethics issues. So, it may be that the female students we surveyed were more likely to view euthanasia as an ethical competence in the early stages of the course, whereas male students may have mainly viewed euthanasia through the prism of clinical competency, and thus viewed it as increasingly important as their graduation drew nearer. Of course, decisions to perform euthanasia are often morally complex and can lead to moral stress in practitioners [17], so the early engagement of students on this topic should be encouraged.

Many animal welfare advocacy and publicity campaigns regarding production animals have focused on welfare issues within intensive production systems. However, the veterinary students we surveyed did not rank the issue of intensive versus extensive production systems as particularly more important compared with the other topics. It is difficult to interpret this response without undertaking further studies, but our finding may reflect the view that both intensive *and* extensive production systems can present animal welfare challenges. Australia and New Zealand have significant extensive livestock sectors, predominantly involving cattle and sheep, with various accompanying welfare issues [18,19]. Veterinary students in these countries are trained to address the challenges that may occur through extreme variations in climate, invasive husbandry practices, and long-distance transport.

Finally, it should be noted that for most of the production AWE topics examined in the current study (ethics of sustainable production; intensive versus extensive production; slaughter and pre-slaughter inspections; social, economic, and cultural drivers; and strategies to address painful husbandry procedures), there were no significant effects of stage-of-study, gender of respondents, or their interaction. Thus, for these topics we did not detect the trends identified in previous studies of declining importance of animal welfare issues for students as they progressed through the veterinary course (e.g., [6]). It may be that as animal welfare becomes a more prominent part of the veterinary curriculum, including being emphasised as part of clinical teaching [20], students may be responding by being more consistent in their ranking of the importance of AWE issues over time [16].

## Figures and Tables

**Table 1:** Schedule for recruiting participants from institutions participating in this survey and response rate.

Institution	Email 1	Closing Date	Number of Students	Number of Responses	Response Percent
The University of Sydney	9 October 2014	7 November 2014	600	147	24.5
Massey University	9 October 2014	7 November 2014	500	141	28.2
James Cook University	10 October 2014	7 November 2014	350	91	26.0
Charles Sturt University	16 October 2014	7 November 2014	295	84	28.5
The University of Queensland	10 October 2014	7 November 2014	609	68	11.1
The University of Adelaide	15 October 2014	7 November 2014	317	119	37.5
The University of Melbourne	17 October 2014	7 November 2014	259 ^a^	52	20.0
Murdoch University	22 October 2014	14 November 2014	390	116	29.7
Total			3320	818 ^b^	24.5%

^a^ Only 1st and 2nd year students surveyed. ^b^ 575 students (70.3%) answered all production animal questions in the survey, but 818 students answered at least 1 question in the survey.

**Table 2:** *p* values obtained from log-linear model on the effect of gender and stage of course on scores to each question, and the interaction of the two factors. *p* values < 0.05 appear in bold font.

Topic	*p* Value for Factors (Degrees of Freedom)		
	Stage (10)	Gender (5)	Stage × Gender (10)
Ethics of sustainable production (food security and animal welfare issues)	0.37	0.07	0.70
Human-animal interactions and impacts on animals	0.03	0.14	0.95
Intensive versus extensive production systems	0.93	0.34	0.65
Slaughter and pre-slaughter inspections	0.07	0.67	0.64
Social, economic and cultural drivers of welfare outcomes	0.23	0.11	0.74
Strategies to address painful husbandry procedures	0.82	0.06	0.40
Distress associated with road, sea and air transport	0.64	0.001	0.33
Euthanasia	0.33	0.32	0.02
